# Maternity Endowments under the Insurance Act

**Published:** 1913-07-26

**Authors:** 


### MATERNITY AND MOTHERING.

**(Inquiries and Correspondence are invited.)**

### Maternity Endowments under the Insurance Act.

Some very instructive comments on the working of the maternity benefit provisions of the Insurance Act, and suggestions for improving various defects in it, are put forward by Miss Davies and Miss Bondfield on behalf of the Women's Co-operative Guild in a long letter to the public press published on July 10, 1913. These suggestions for amendment of the Act are endorsed by the Association of Approved Societies; and although we cannot agree with everything that the two ladies state in their letter, undoubtedly their criticisms are for the most part justified and their suggestions helpful.

The first contention of the Women's Co-operative Guild is that the thirty shillings maternity benefit, which is paid because the husband is an insured person, should be legally the wife's property and payable directly to her, not to the husband. Cases have come to light, and no doubt many more have arisen without coming to light, in which the possession of this thirty shillings has tempted the husband into an immediate debauch; so that the unfortunate wife, for whose benefit during the lying-in period it is intended, never gets the money at all, or only a small fraction of it. In one case, it is said, the husband used the money to go away with another woman, a particularly cruel instance of depravity.

With this proposal we find ourselves in entire sympathy. It is, of course, true that there are married men so dead to all sense of shame that they would actually take the money from their wives at the first opportunity. No safeguards will circumvent such ruffians. But these extreme cases are very rare, and not nearly so common as those in which a weak man, suddenly possessed of thirty shillings he has not had to earn, is unable to refrain from celebrating the fact by alcoholic excess with his friends. In the great majority of cases, once the wife got possession of the money, she could be relied upon to retain it, and put it to proper uses. It must not be overlooked that in a certain number of cases it might be—undoubtedly would be—the wife that would succumb to the attractions of the public-house. But that again would be exceptional; and we think a case has been made out for this particular reform.

The next provision of the Act which Miss Davies and Miss Bondfield would have revised is that which allows a doctor, called in by a midwife to attend an insured man's wife owing to some obstetric emergency, to be paid his prescribed fee (usually one guinea) out of the maternity benefit. This clause they wish to see struck out altogether, but the arguments they bring forward in favour of this course are pitifully weak and unsubstantial. They say that the wording of the clause leaves it uncertain whether the liability to the doctor attaches to the approved society, the Insurance Committee, or the insured person. Remedy the uncertainty, by all means; but that is no argument for repealing the whole clause. It is further argued that this provision makes against the woman's free choice between doctor and midwife, a statement so absolutely ridiculous on the face of it that it shows up the weakness of the whole case. The woman to whom this clause applies is one who has already exercised her free choice in favour of a midwife, but is in urgent need of immediate medical skill owing to some desperate emergency with which the midwife is unable to cope. To throw difficulties in the way of her swift succour in such an event is not exactly to her advantage. "There is no way out of the mess," say the two ladies, but the whole of the evidence they bring forward leads to an exactly opposite conclusion. On this point, therefore, we cannot support their claim. Furthermore, a scheme to get over the whole difficulty has been evolved by the English Commissioners and is set forth in detail on page 506 of this issue.

The third point raised is that of confinement sick pay. By dint of her husband's contributions, a married woman is entitled to thirty shillings on the birth of each child. Should she herself be an insured worker also, she is further entitled to sick benefit at the rate of seven and sixpence a week for four weeks; that is, to a further thirty shillings. The object of this is to prevent her premature return to her employment, for this seven and sixpence a week is only provided on condition she does not do this, and an excellent idea it is. It appears, however, according to Miss Davies and Miss Bondfield, that some societies are refusing to pay unless satisfied on medical evidence that the mother is unable to work at her employment. Obviously, this will have the effect of driving insured mothers back to work a fortnight or so after their confinements, the very evil which the Act sought to remedy.

We cannot believe that the Act is so badly drafted as to afford shelter to the societies for such a scandalous misinterpretation of its intentions; nor can we understand how the management of any approved society could advance such a technical excuse for these acts of unconscionable harshness, even if it really does exist. The disclosure of this monstrous injustice is surely sufficient to prevent its perpetuation; but to make assurance doubly sure it would be well to add a passage to the amending Bill to clear up any possible doubt on the matter. If this is the way the societies behave to their own members, it is easy to see why the medical men object to being put under their thumb; and the lesson will not be wasted, in view of the agitation going on among the friendly societies to regain control of medical benefit.

